# Female empowerment to improve sexual and reproductive health outcomes and prevent violence in adolescent girls and young women in Uganda: evidence reviews for policy

**DOI:** 10.4314/ahs.v22i4.47

**Published:** 2022-12

**Authors:** Jessica Lewington, Rosemary Geddes, Grace Gabagaya

**Affiliations:** 1 Centre for Population Health Sciences, University of Edinburgh, Edinburgh, UK; 2 African Centre for Migration & Society, University of the Witwatersrand, South Africa; 3 Makerere University-Johns Hopkins University Research Collaboration Affiliation, Kampala, Uganda

**Keywords:** Sexual health, health policy, women's health

## Abstract

**Background:**

Adolescent girls and young women in Uganda face numerous public health challenges including high HIV prevalence, teenage pregnancies, poor sexual and reproductive health rights, child marriage, and violence.

**Objectives:**

This evidence review explores which interventions focusing on the empowerment of adolescent girls and young women to address these challenges are suitable for Ugandan policy.

**Methods:**

We reviewed the literature to identify experimental studies and systematic reviews of interventions which improve sexual and reproductive health outcomes and/or prevent violence in adolescent girls and young women in low- and middle-income countries (LMICs). Two authors independently reviewed the studies identified through a comprehensive search strategy and assessed their quality. From this evidence base, two policy options were explored in depth considering benefits, harms, equity impacts, and costs, given the Ugandan context.

**Results:**

The screen yielded 47 studies, of which 12 remained after applying inclusion and exclusion criteria and relevance, applicability and quality assessment. Feasible policy options included: a vertical cash-incentive approach at a national or local level to support girls' attainment of education; and a horizontal integrated community approach focusing on skills and knowledge building. A combination of both is recommended for young female empowerment in Uganda, allowing for the full range of socio-cultural and economic drivers to be targeted.

**Conclusion:**

Research into the link between female empowerment and sexual and reproductive health outcomes is still in early development. This review contributes to evidence on this topic and outlines an approach that is potentially suitable for adoption across similar LMICs in Africa.

## Introduction

There are an estimated 580 million adolescent girls worldwide, 88% of whom live in low-and-middle income countries (LMICs) where sexual and reproductive health (SRH) is a major issue^1^. Adolescent girls and young women are frequently denied the right to make decisions about their own health and wellbeing^2^, and the targets of domestic, partner and emotional violence and child marriage^3^–^9^. In Uganda, like many LMICs, young women face SRH challenges and are often the victims of violence including intimate partner violence^10^;^11^. Although Uganda has made strong progress in reducing the prevalence of HIV/AIDS, with a reduction from 15% in 1990 to 5.7% in 2018 (15–49 years), HIV/AIDS is still unequally distributed with a higher prevalence in women than men (8.8% vs. 4.3%)^12^. Uganda retains a high burden of other SRH risks such as teenage pregnancy, with 33% of Ugandan women having given birth before the age of 18 years which is among the highest in the world^13^. Maternal mortality is the leading cause of death among women of childbearing age in Uganda with more than 80% of all maternal deaths a result of preventable causes^14^. Sexual violence affects more than one in five women in Uganda, with UNFPA Uganda reporting 22% of women aged 15–49 experience sexual violence at some point in time^15^. Reports estimate that as many as 60% of women have experienced gender-based violence in Uganda^16^. Furthermore, a study of 15–19-year-old sexually experienced girls in Rakai, Uganda, found that 14% of the 575 girls had experienced coerced first sexual intercourse^3^. The consequences included unintended pregnancies, unsafe abortion, sexually transmitted diseases (STDs), an inability to negotiate safer sex and protective behaviours, and inability to affect their partners' risk-taking behaviour.

Socio-economic, structural and cultural determinants drive these issues. Young girls are more likely to drop out of school and marry at an early age. An estimated 40% of girls in Uganda marry before their 18^th^ birthday^17^. This is despite the Children's Act 2016 stipulating the minimum legal age of marriage as 18 years for both sexes^18^, as well as Uganda ratifying the Convention on the Rights of the Child in 1990, which sets a minimum age of marriage of 18 years^19^. Child marriage has many health effects, including an increased risk for STDs, cervical cancer, malaria, death during childbirth, and obstetric fistulas^20^. In rural areas of Uganda in particular, cultural norms legitimise, promote and accommodate early marriages^11^. Cross-generational and transactional sexual relations, affecting an estimated 11.8% of adolescent girls in Uganda, put them at heightened vulnerability to: STDs (including HIV); unwanted and risky early pregnancies; unsafe abortions and the associated higher risk of maternal morbidity; injury or trauma of the reproductive system when young and physiologically immature; and increased likelihood of being engaged in trafficking or the sex trade^21^. Drivers of cross-generation and transactional sex include economic, social, and structural factors characterised by poverty, discriminatory social norms, high-risk physical environments, and lack of access to quality community resources and essential services. In rural areas, physical distance to facilities including school means girls sometimes exchange sex for transportation 11.

Greater gender equality and female empowerment are associated with reduced risk of intimate partner violence, improved general health, fertility, and mortality outcomes, better access to education, and greater economic security^22^–^24^. Ugandan policies support the improvement of SRH outcomes for adolescent girls and young women^25^. These include: 1997 National Gender Policy to address gender inequalities and promote the empowerment of women and women's health; 2004 Adolescent Health Policy; and 2006 National Policy Guidelines and Service Standards for Sexual and Reproductive Health and Rights. However, policy impacts on the SRH needs of young people has been less than adequate^12^, as acknowledged in Uganda's Vision to 2040 plan in 2013, with gender inequality and sexual and gender-based violence both highlighted. During the UN Universal Periodic Review for Uganda in 2011, the government accepted recommendations to reduce the high maternal mortality rates and increase access to SRH services by raising the health budget to 15%^26^. Uganda, however, failed to meet its female empowerment and reproductive health MDGs^27^. The Committee on Economic, Social and Cultural Rights recommended intensified efforts to reduce the maternal mortality ratio by adequately equipping facilities to provide maternal and postnatal care^28^. Despite this, high maternal mortality remains with health expenditure at 7.3% in 2015 and 6.5% in 2018^29^.

A number of initiatives aim to reduce the vulnerabilities of adolescent girls, focusing on how economic empowerment interventions such as waivers of school fees, cash transfers and microfinance schemes can allow vulnerable girls in LMICs to be less reliant on male partners^30^. Similarly, research into ways to prevent child marriages in LMICs suggests that empowering girls and offering incentives can be effective^29^. There is limited research, however, on whether these programmes are sustainable and feasible. This evidence review for policy explores whether interventions focusing on the empowerment of adolescent girls and young women to improve SRH outcomes and prevent violence are suitable for Uganda.

## Methods

We drew on Supporting the Use of Research Evidence (SURE) guides for evidence-based policy briefs^31^, as recommended by the WHO^32^. This included: clarifying the problem using appropriate tools; systematically reviewing the literature; and reviewing the context in Uganda to assess the feasibility of local implementation.

### Search strategy

A comprehensive literature search strategy was constructed to identify studies reporting on female empowerment interventions to improve SRH outcomes and prevent violence against adolescent girls and young women. Databases searched were: PubMed (2005–2019); Global Health (CABI) (2005–2019) and Cochrane (2005–2019). Details of search terms and the population, intervention, control, outcome (PICO) search strategy is provided in the Appendices (A; B).

### Selection criteria

Experimental or quasi-experimental studies or systematic reviews (SR) that evaluated programmes reporting on female empowerment interventions to improve SRH outcomes and prevent violence against adolescent girls and young women were sought. Inclusion criteria were: published studies in English; interventions implemented in adolescent girls and young women (age 10–24 years) in LMICs; interventions focusing on empowerment approaches; and outcomes including SRH and/or preventing violence against women. Empowerment approaches included life skills and economic interventions at the national, household or individual level, as long as they were specifically targeted at adolescent girls and young women. SRH outcomes included those relating to sexual risk behaviour, marital status, pregnancy and births, and sexually transmitted infections. We excluded studies: with females of other age groups; that were non-gender specific; in high income countries; with interventions that focused on women's health issues without an empowerment approach; and focusing on non-health related development outcomes.

### Applicability, quality assessment and data extraction

One author conducted the initial screen of paper titles and abstracts. Two authors independently reviewed the retrieved full papers that met the inclusion criteria, resolving selection disagreements through discussion. Selected papers were assessed for applicability to the Ugandan context using a checklist adapted from SUPPORT Tools for evidence-informed health Policymaking^33^ which includes questions related to setting, time period, resource constraints, health system, and key baseline conditions. Two authors independently assessed the quality of the remaining papers, using Critical Appraisal Skills Program^34^ checklists for SRs and randomised controlled trials (RCT), and a checklist adapted from the Joanna Briggs Institute for quasi-experimental studies^35^. One author extracted data on: study design; setting; target population; intervention description; provider and funding source; study outcomes; and results (Appendix C).

From this evidence base, we explored potential policy options according to a framework developed by Buffet et al.^36^ for use in Uganda. Key criteria for feasibility included political and social acceptability, availability of resources, organisational expertise and capacity, and transferability from the country where the intervention was tested. Furthermore, the WHO's SURE framework^31^ was adapted to identify potential barriers and enablers to implementation, categorised across four levels: recipients of care; providers of care; health system; and social and political. To address barriers, a rapid literature review was conducted using the Cochrane Database of Systematic Reviews, PubMed, Health Systems Evidence and SUPPORT summaries, and grey literature. Finally, local stakeholders were also consulted to discuss policy options and ways to overcome barriers.

## Results

**Figure 1:**
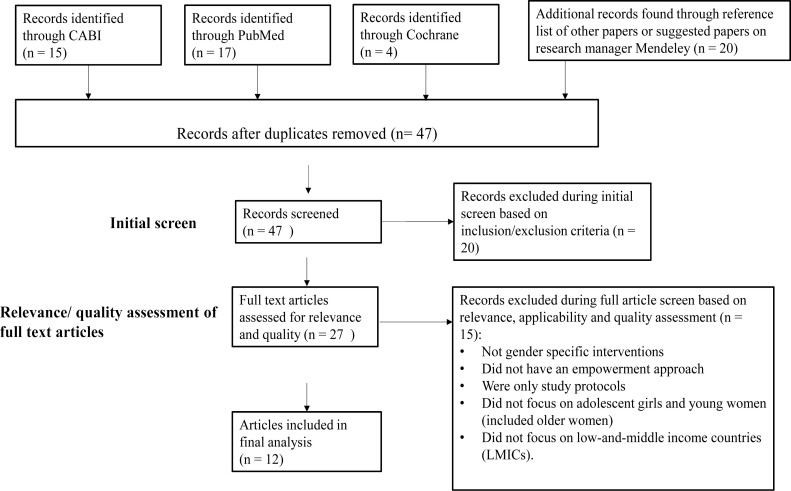
PRISMA flow diagram

The literature search yielded 47 papers. On review of titles and abstracts, 27 papers were relevant based on inclusion criteria. The full text of these were assessed in detail for relevance, applicability and quality, after which a further 15 papers were rejected. Twelve studies were included in the final analysis (PRISMA diagram Figure 1), including six RCTs, four quasi-experimental studies and two SRs, all of which were from LMICs. The studies were of moderate to high quality and showed positive outcomes in most settings, with some showing mixed results. Follow-up was for one to two years in most studies. The types of interventions included in the primary studies were categorised as: vertical, cash-incentive approaches either at national, district or individual level; and horizontal, integrated, and multi-component approaches that include skills and knowledge building. The two SRs were categorised as mixed as they include studies from both of these approaches.

The cash transfer interventions at individual or household level based on school-enrolment led to significant increases in school enrolment and attendance, and reductions in school dropouts in all settings, except South Africa where school attendance was already high for both intervention and control groups^37^ (Table 1 and Appendix C). Furthermore, in South Africa, there was significantly less intimate partner violence, unprotected sex, and women had fewer sexual partners in intervention groups^37^. In Malawi there was significantly lower HIV and HSV-2 prevalence in intervention groups at follow-up, with weighted prevalence of HIV was 1·2% in the intervention group versus 3·0% in the control group at 18-month follow-up (OR 0.36 95%CI)^39^. A national level unconditional cash transfer (not requiring minimum school attendance but meeting other vulnerability criteria; Table 1) in Kenya led to significant reductions in pregnancies but not marriage^40^. In Pakistan, intervention group girls were significantly more likely to delay marriage^38^.

**Table 1:** Cash transfer programmes suitable for LMICs with mechanisms, recipients, results and parameters for usage

Cash-transfer programme	Mechanism of cash-transfer	Parameters for cash usage	Recipient with responsibility for the cash-transfer	Main beneficiary	Key results of study and effectiveness
Government led cashtransfer programme in Punjab, Pakistan (38)	A quarterly stipend of approximately PKR 600 (equivalent to US$10 in 2011) per female student in grades 6 through 8. Beneficiary girls were targeted based on their district of residence (districts with the lowest literacy rates in the province – below 40%). Eligibility was conditional on a minimum school attendance rate of 80%, as reported by the school.	School enrolment with cashtransfer conditional on a minimum school attendance rate of 80% as reported by the school. Leftover funds were allowed to go towards general family support.	Not disclosed.	Adolescent girl.	Early evaluation of the programme shows that the enrolment of eligible girls in middle school increased in the short term by nearly 9%. Girls in stipend districts were likely to delay marriage by about 1.2–1.5 years. Relative to the control group, girls exposed to the programme have on average 0.3 fewer children. However, significance of this is only at the 10% level.
National cash-transfer in Kenya (40)	Cash transfer of 1500 Kenyan shillings ($USD 22 in 2015), which comprises nearly 20% of average monthly total household expenditure, was distributed to eligible households for use towards schooling costs. Eligibility required	Not specified beyond towards schooling costs.	Parent/guardian with funding received at household level.	Orphaned or vulnerable adolescent girls.	Significant difference in pregnancy status (19% control versus 13% treatment; p 0.01) but not for marriage. This is lower than the cash transfer programme in South Africa (see below) which had a difference of 10.5% (37).
Cash-transfer programme in Zomba district of Malawi (39)	The programme beneficiary and her parents/guardians were made an offer that specified the monthly transfer amounts offered to the beneficiary and her parents, the condition to regularly attend school, and the duration of the programme. Cash transfer was split between parent/guardian of adolescent girl and the adolescent girl (programme beneficiary) herself. Parents/guardians and adolescent girls visited cash-transfer points to collect money.	Not specified beyond schooling costs, with adolescent girls receiving funds on condition of regular attendance.	Split between adolescent girl and parent/guardian.	Adolescent girl.	Intervention effects on primary outcomes assessed for 1706 individuals. In the cohort of baseline schoolgirls, weighted prevalence of HIV was 1·2% in the intervention group versus 3·0% in the control group at 18- month follow-up. Weighted HSV-2 prevalence was 0·7% in the intervention group versus 3·0% in the control group. There was a significant difference in HIV prevalence between schoolgirls in intervention group versus control group (OR 0.36 95%CI) and for HSV-2 prevalence between these groups (OR 0.24 95% CI).
Cashtransfer in rural South Africa (37)	Women aged 13–20 years who were enrolled in school grades 8–11 of the South African educational system had to be able to open a bank account as well as their parent/guardian. Young women randomly assigned to the intervention group received 100 rands ( $USD 10 in 2012), and their parent or guardian received R200 (about $USD 20) every month, conditional on the young woman attending 80% of school days per month.	Not specified beyond schooling costs, with adolescent girls receiving funds on condition of regular attendance (80%).	Split between adolescent girl and parent/guardian, with parent/guardian receiving double	Adolescent girl	No significant difference was recorded in HIV incidence. However, participants who received the cash transfer were significantly less likely to report experiencing partner physical violence in the past 12 months (18% in treatment group and 28% in control group), to have had a sexual partner in the past 12 months, and to report engaging in unprotected sex in the past 3 months than were those in the control group. Physical violence from a partner in the past 12 months has risk ratio 0.66 (0.59 to 0.74) with p<0.0001.
Cash- transfer programme in rural eastern Zimbabwe (41)	Cash-transfer programme was organised directly with the schools involved in the programme and funds were specific to school fees, exercise books, uniforms and supplies for each student. Additional funding was provided to pay for the school-based helpers and for the food programme.	Intervention students received school support including fees, exercise books, uniforms, and other school supplies (e.g., pens, soap, underpants, and sanitary napkins), and a school-based helper to monitor attendance and resolve problems. In addition, all primary schools involved received a universal daily feeding programme.	Schools are responsible for the funds intended for eligible students.	Adolescent orphan girls	Compared with intervention participants, control participants had 6 times the odds of dropping out of school and nearly 3 times the odds of getting married after 2 years. Reduced HIV risk was calculated through delaying early marriage. The intervention reduced school dropout by 82% and marriage by 63% after 2 years. For school dropouts, control group vs treatment group OR = 8.5, p .0.001; for getting married control group vs treatment group OR=2.92, p=0.2. Treatment group also more likely than were control participants to endorse gender equity (p=0.07) and were more likely to report waiting for sex because of the consequences (p=0.03).

The integrated, multi-component approaches to empower adolescent girls including training, knowledge- and skill-building, and mentorship within the community. Two also included a micro-loans initiative, one a savings account and one basic training in managing finances (Table 2 and Appendix C). Studies were based in Kenya, Uganda and Zimbabwe. Three studies reported significant increases in contraception use and decreases in unwilling or transactional sex^42^;^43^;^45^. Two studies reported a significant increase in knowledge of HIV, reproduction and pregnancy^42^;^43^. One study also reported a significant decrease in childbearing^43^.

**Table 2:** Key results and effectiveness of horizontal, integrated multi-component studies assessing empowerment interventions to improve sexual and reproductive outcomes in LMICs

Horizontal, multi- component programme	Key results of study and effectiveness
The Nairobi TRY programme (42)	Chi-square tests showed – For gender attitudes: • Significant difference between baseline and endline in attitude towards refusing sex (33.2% to 48.1%, p<0.001) • Significance at 10% level for disagreeing that marriage is required if a girl is unschooled (80.9% to 86.0%) and needing a husband to be happy (46.0% to 51.8%) Changes in reproductive health knowledge: • Significance in knowledge shift on HIV for a few of the outcomes (not all) at p<0.05 level. Sexual negotiation and decision making: • Able to insist on condom use with spouse/partner (55.8 to 61.7; p<0.01) • Take part in contraception decision making in general (96.3 to 98.0; p<0.05).
Rape Prevention Through Empowerment of Adolescent Girls in Nairobi (44)	Adolescents who underwent training in assault prevention strategies were more able to protect themselves from sexual assault and harassment, and more likely to disclose assaults that did take place, than those who did not receive training. The rate of sexual assault decreased from 17.9 to 11.1 per 100 person-years in the intervention (p < 0.001), as opposed to no significant change in the SOC group.
The Empowerment and Livelihood for Adolescents (ELA) programme in Uganda (43)	Both HIV knowledge and pregnancy knowledge raised significantly. Self-reported condom usage increased by 50%, and probability of having a child decreased by 26%. Girls who reported having sex against their will dropped from 21% to 0%. Intention to Treat (ITT) analysis saw increase in HIV knowledge significant p<0.01; increase in pregnancy knowledge p<0.05; increase in using condom if sexually active p<0.05; reduction in childbearing p<0.05; and reduction in sex unwillingly p<0.01. Gender empowerment index increased significantly p<0.01.
Shaping the Health of Adolescents in Zimbabwe (SHAZ!) (45)	RCT of a combined intervention package including life-skills and health education, vocational training, micro-grants and social supports, compared to life-skills and health education alone. Adolescent females in study arm had lower risk of transactional sex [IOR = 0.64, 95% CI (0.50, 0.83)], and a higher likelihood of using a condom with their current partner [IOR = 1.79, 95% CI (1.23, 2.62)] over time compared to baseline. Also, fewer unintended pregnancies among intervention participants [HR = 0.61, 95% CI (0.37, 1.01)], although this relationship achieved only marginal statistical significance.
Social, health and economic assetbuilding intervention for vulnerable adolescent girls in Uganda (46)	Quasi-experimental study with two intervention groups and a comparison group. The first treatment group received the full intervention . safe spaces group meetings with reproductive health and financial education plus savings accounts . while the second group only received a savings account. The full intervention was associated with improvement in girls' health and economic assets. Girls who only had a savings account increased their economic assets but they were also more likely to have been sexually touched (OR = 3.146; p<0.01) and harassed by men (OR = 1.962; p<0.05).

### Policy options to improve SRH, empower adolescent girls and young women and prevent violence

Two policy options were explored: a cash-incentive approach at a national, district, household or individual level to support girls' attainment of education; and an integrated multi-component community approach focusing on skills and knowledge building.

### Policy option one: Cash-incentive approach

Of the ten primary studies, five focused on a cash-transfer element and were either district, regional or national interventions (Table 1). This was also a key approach in one SR on preventing child marriage^4^. This policy approach aims to invest in education and empower adolescent girls by providing financial support thereby removing the link between poverty and negative SRH outcomes. This was also a key approach in one SR on preventing child marriage^4^. In the majority of the cash-transfer programmes, funds were given to individuals or households based on school enrolment (and in some cases minimum attendance) or a female adolescent in the household. In one intervention, funds went directly to the schools to provide supplies for adolescent orphan girls^41^. These interventions are in response to evidence which suggests a strong link between duration of school attendance and a reduced risk for HIV, child marriage and other SRH challenges. In South Africa, adolescent girls and young women aged between 15–24 years were found to be three times less likely to have HIV if they completed schooling^47^. Similarly in Botswana, each additional year of secondary schooling led to an absolute reduction in the cumulative risk of HIV infection^48^.

Despite the evidence that keeping girls in school for longer reduces their risk of HIV infection, barriers exist for them to attend school. These include school fees, cost of school uniforms, family and domestic responsibilities, and societal norms which are not supportive of girls' education^37^.

National-level cash transfer approaches also showed effectiveness; the Kenya programme demonstrated success on a large-scale, impacting positively on secondary school enrolment and therefore preventing early pregnancy^40^.

### Policy option two: Int e grated multi-component approach with a focus on skills and knowledge building

Five identified studies, two in Kenya, one in Zimbabwe and two in Uganda, took a more horizontal, integrated approach in either the school or community setting (Table 2). The Nairobi TRY programme^42^ took a multi-component approach including a cash-transfer intervention combined with mentorship, skills and knowledge building to empower adolescent girls and young women. Similarly, the Empowerment and Livelihood for Adolescents (ELA) programme^43^, conducted in Uganda, incorporated life skills to build knowledge and reduce risky behaviours as well as vocational training enabling girls to establish small-scale enterprises (but did not include a cash-transfer element). The ELA programme saw ‘adolescent development clubs’ established within each community, where key topics such as SRH, menstruation, pregnancy, STIs, HIV/AIDS awareness, family planning, and rape were covered. Other sessions covered enabling topics such as management skills, negotiation and conflict resolution, leadership among adolescents, and legal knowledge on women's issues such as bride price, child marriage and violence against women. A second programme in Nairobi centred on adolescent girls being taught empowerment and self-defence skills to assess the impact on the incidence of sexual assault and harassment^44^. The Shaping the Health of Adolescents in Zimbabwe (SHAZ!) provided a package including life-skills and health education, vocational training, micro-grants and social supports, compared to life-skills ad health education alone^45^. The study arm participants had a significantly lower risk of transactional sex, and a higher likelihood of using a condom with their current partner over time compared to baseline. Finally, the Social, health and economic asset-building intervention for vulnerable adolescent girls in Uganda provided safe spaces for group meetings with reproductive health and financial education plus savings accounts, the second group receiving only a savings account^46^. The full intervention was associated with improvement in girls' health and economic assets, whilst girls who only had a savings account increased their economic assets but they were also more likely to have been sexually touched and harassed by men (Table 2).

### Feasibility and acceptability

The cash-transfer studies included were all conducted in LMIC settings, thus transferable to a Ugandan context, with four studies conducted in Africa and one in Pakistan. Providing incentives for education is seen to be politically popular and of interest to large-scale actors such as educational systems and multi-lateral organisations in LMICs^4^. This approach, particularly where objectives relate to school enrolment, aligns with a number of government policies in Uganda^49^. Although programmes based on school attendance and enrolment may be acceptable and can prevent financial barriers, feasibility is likely to be more complex, due to challenges encountered with actual school completion in Uganda. Local evidence shows a number of social and cultural barriers exist for adolescent girls to attend school, including school location, menstruation, cultural beliefs, sexual abuse and exploitation within the school environment, lack of income-earning possibilities, and domestic responsibilities^50^;^37^. Moreover, the success of this policy in the school setting depends on girls being targeted at the appropriate age, before school attendance declines dramatically. The Ugandan Demographic and Health Survey 51 shows the reduction in school attendance by increasing age in urban Uganda (Table 3). For ages 10 and 11, only 4.1% are out of school; for girls aged 12 this more than doubles to 9.5%; and by 14–17, nearly half (43.2%) of girls are no longer attending school. For rural areas figures are similar, with slightly more girls still in school (32.9% no longer attending) from age 14–17 (ibid). Therefore, cash-transfer programmes should be implemented before age 13 or they would miss the critical age girls leave school. Alternatively, complementary incentives to keep girls in school for longer would be needed.

**Table 3:** Reduction in school attendance by age (%), Source: DHS Survey 2011 cited by UNESCO, 2013

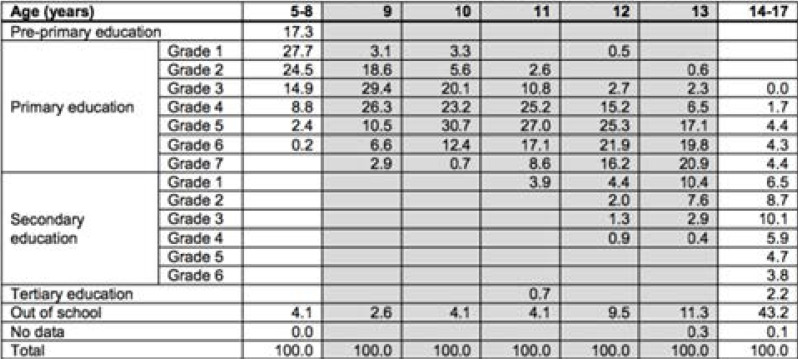

Resources to support adolescent girls through a cash-transfer programme need to cover school fees, books, school uniforms, supplies and transport. The majority of the cash-transfer programmes assessed in this review were funded by NGO grants. Only two^38^;^40^ were funded by Government, however, a range of funding sources should be considered in the LMIC context. Furthermore, longer-term financial sustainability of these approaches should be considered, including sustained benefits to grantees. Uganda's Youth Opportunities Programme, which granted US$400 per person to start skilled trades, found positive results after four years, but after nine years grantees' investment levelled off and limited impact was made on fertility and health^52^. However, given the strong evidence showing that school attendance delays marriage and improves SRH outcomes^38^;^41^;^39^;^4^;^37^, by receiving an education alone, a sustainable impact on girls' lives is possible. Evidence suggests that by empowering adolescent girls and young women, Uganda would achieve long-term financial gains. The World Bank estimates that ending child marriage in Uganda could generate US$3 billion per year for Uganda's population by 2030^53^. The reasons for this include: girls receive higher education and enter the job market; a reduction in costs associated with providing services for teenage pregnancy and its complications which puts pressure on the national budget; and reductions in maternal mortality and morbidity^54^. Reductions in maternal mortality alone have accounted for 11% of recent economic growth in LMICs^55^.

With regard to policy option 2, the primary studies were all conducted in LMIC settings, including two in Uganda, and are thus transferable. Success of the ELA programme in Uganda shows a “latent demand”^43^ for such programmes within the community, with adolescent girls and young women volunteering to join and undertake training. A number of other integrated, multi-component programmes across Uganda not yet reported in peer-reviewed literature are also successfully running, demonstrating an appetite for local initiatives. These include: the Uganda For Her initiative, which teaches SRH and rights to encourage respect and help abolish SRH stigmas or myths^49^; the Determined, Resilient, Empowered, AIDS Free, Mentored, and Safe (DREAMS) Project, which aims to reduce new HIV cases in women aged 15–24 by addressing the structural drivers, including poverty, gender inequality, sexual violence and lack of education^56^; and Forum for African Women Educationalists programmes, which aim to accelerate female participation in education through scholarships accompanied by SRH education components and female mentoring^57^.

Four identified studies were conducted in the community setting, and one was school-based. As mentioned earlier, numerous policies in Uganda support school-based programmes and empowerment to improve SRH for adolescent girls, and align with community-based approaches. By taking a more integrated, grassroots approach within the community, empowerment programmes can be designed cognisant of the unique challenges faced by adolescent girls and young women in the area. This community driven approach also offers the benefit of being more sustainable 4. However, this nuanced style also means that the ability to scale may be more difficult.

### Implementation – barriers and enablers

Recipient unawareness of eligibility for empowerment programmes could lead to low uptake. Community involvement has been found to enhance community ownership, increase transparency, and reduce potential for conflict and jealousy^58^. Lack of recipient knowledge to manage money or set-up bank accounts is a further barrier. This can be overcome by issuing funds wholly to the parent/guardian, splitting between the parent/guardian and the adolescent girl, or issuing to the school to cover all schooling costs for enrolled students^41^;^39^;^40^;^37^. However, basic budgeting skills should be provided to those responsible for receiving the cash-transfer. Stakeholders recommend that people with the right technical skills work collaboratively with the Ugandan Government to do needs assessments to appropriately target adolescent girls and young women.

Ensuring there is adequate knowledge and skills on the ‘provider’ side is integral to the success of the community-based component. To build in a multi-component approach that includes training, skill-building and mentorship, local mentors from within the community across a range of professions will need to be available and trained. There is strong evidence that ‘Train the trainer’ (TTT) approaches can serve as mechanisms to disseminate knowledge and skills in low resource settings^59^. Uganda-4Her's SRH programme uses the TTT method for ‘peer educators’ to conduct the SRH sessions and in turn to train others to carry on this work. They also use girls' clubs at school, where students and staff members are selected for SRH training, so that learning can be shared sustainably throughout the school community. However, appropriate supervision and monitoring of performance is needed to ensure that cascading knowledge is not impeded by factors like attrition^60^.

Accessibility to both schools and community centres need consideration for both policy options. The Universal Primary Education Policy^61^, provides accessible school infrastructure with the appropriate level of coverage in Uganda. These facilities, more accessible to girls, can be used for the community component of the intervention, particularly in rural areas where access to health and community centres is sparser. The ELA programme in Uganda^43^ was designed using ‘adolescent development clubs’ rather than in schools, and reached school drop-outs as well as girls currently enrolled in school. Community centres could therefore be used as additional options.

Financial and human resources must be considered to scale nationally. For example, the ELA was NGO-run and had very frequent interaction and high asset transfer amounts. It may be difficult to reasonably implement the intensity of the inputs at scale. However, the International Labour Organisation argues that basic social protection benefits are not unaffordable in LMICs in Sub-Saharan Africa, even though some international assistance would be necessary for a transitory period^53^. Health worker shortages present a challenge for the community skills and knowledge policy option. To improve SRH and child mortality as part of the MDGs, the WHO recommended the use of task-shifting to community health workers^62^. Uganda benefits from already having an organised lay health worker system, the Village Health Teams, introduced as part of Uganda's National Health Strategy in 2001 to improve access to services, and serving as the primary, village-level health contact to relay basic health information^25^. Similar approaches could be used to train community members to run community ‘adolescent development clubs’ such as in the ELA programme.

Information systems are also crucial to monitoring uptake and distribution of the cash-transfer component. Uganda launched a national Health policy and strategy 2017–2021 in May 2018 to strengthen digital infrastructure and eHealth capabilities.

A strong social and cultural stigma around SRH still pervades in Uganda and is a key barrier to women seeking out key services^63^. However, a SR found that young people, particularly those at risk for HIV and reproductive health-related problems, were more likely to seek help at youth centres than traditional facility-based health services^64^. Interventions implemented in school and community settings, are similarly likely to promote health-seeking behaviour from adolescents.

Political will is crucial for successful implementation in Uganda, particularly cash-transfer programmes which require substantial financial input for national scaling. Beyond national legislation, the Ugandan Government has pledged improvements in SRH on the international stage by committing to the SDGs. They were one of the first African countries to develop their 2015/16–2019/20 National Development Plan in line with the SDGs and partnered with international organisations to try and achieve this^27^. Cash transfer funds are potentially vulnerable to fraud and corruption, however numerous approaches exist to reduce security risks^65^;^66^.

## Discussion

This paper explores two policy options for implementation in Uganda: a cash-incentive approach; and an integrated multi-component approach with a focus on skills and knowledge building. Barriers and enablers at the recipient and provider levels, the system level and the social and political level were considered. The authors recommend interventions to be initiated before age 13 in girls to best target girls before they are found to typically leave school. Additionally, social and cultural factors need to be addressed, where strong community involvement including local training, skill-building and mentorship within the community enabling this. Resources are a major consideration and substantial government funding would be required for sustainability. However, as the majority of the cash-transfer programmes assessed in this review were funded by NGO grants, a range of funding sources should be considered in the LMIC context. Additionally, this paper's literature review demonstrates convincing evidence that initial investments would be outweighed by long-term economic benefits. The above policy options are aligned with current Ugandan policy, which explicitly prioritises and encourages gender equity and female empowerment, and with Uganda's SDG commitments.

Limitations of this evidence review include the paucity of research in this area. Work to expand current primary research interventions into empowerment interventions effective for young women and girls in LMICs, as well as robust systematic reviews of these interventions, is strongly recommended. Furthermore, by limiting this review to interventions focusing only on adolescents and young women, interventions targeting older women were excluded, but may have been effective in younger groups. We also did not explore the important role of men in supporting female empowerment or of non-gender specific interventions, however, including these elements may make it difficult to specifically identify what is working. Further research into mixed-gender approaches to empower girls and breakdown cultural norms, particularly around SRH and IPV, is also recommended.

## Conclusion

This review adds to the growing evidence which suggests both cash-incentive interventions at a national or local level to support girls' educational attainment, and integrated community approaches focusing on skills and knowledge building, can empower adolescent girls and young women in LMICs to improve SRH outcomes and prevent violence. This paper found a combination of both strategies to be most effective, and the author's recommend that both approaches be adopted into policy in the Ugandan context allowing for the full range of socio-cultural and economic drivers to be targeted. The need for health policy and programming specific to the unique needs of adolescent girls and young women is increasingly being recognised on the international stage as not only achieves improvements in SRH, but is crucial to progressing a nation's overall development. This paper provides much needed evidence for policymakers in Uganda and wider LMIC settings to uplift girls and young women in society in line with crucial and timely SDG Goals.
